# Prevalence of risky sexual behaviors and associated factors among students at a private university in Lebanon

**DOI:** 10.3389/fpubh.2025.1678926

**Published:** 2025-12-19

**Authors:** Youssef Rizk, Michele Cherfane, Wassim Arnaout, Ali El Tannir, Jihane Naous, Rania Sakr

**Affiliations:** 1 Division of Family Medicine, Department of Internal Medicine, Lebanese American University Gilbert and Rose-Marie Chagoury School of Medicine, Beirut, Lebanon; 2 Lebanese American University Gilbert and Rose-Marie Chagoury School of Medicine, Byblos, Lebanon; 3 Department of Community Health and Family Medicine, University of Florida College of Medicine, Florida, FL, United States

**Keywords:** risky sexual behavior, social media, drug use, oral sex, university students

## Abstract

**Background:**

Engaging in unsafe sexual practices remains a major public health concern among young adults worldwide. Despite extensive global research, there is limited evidence from the Middle East, where sexuality is often a taboo subject and sexual education is minimal. This study aimed to examine the prevalence of risky sexual behaviors among university students in Lebanon and to identify factors associated with these behaviors, including substance use and engagement with social and digital media.

**Methods:**

A cross-sectional online survey was conducted among undergraduate and graduate students at the Lebanese American University between May and November 2022. Participants completed an anonymous, self-administered questionnaire assessing sociodemographic characteristics, sexual history, and multimedia use. Data were analyzed using IBM SPSS Statistics version 29. For the count outcome, poisson regression was applied to identify factors associated with a higher number of risky sexual behaviors, defined as unprotected sex, multiple simultaneous partners, sexual activity under the influence of substances, or transactional sex.

**Results:**

Among 588 participants, 233 (39.6%) were sexually active, of whom 225 (96.6%) reported at least one risky sexual behavior, with a mean of 2.24 risky behaviors per participant. Unprotected oral sex was the most common unsafe practice. Students who reported drug use were significantly more likely to engage in a higher number of risky sexual behaviors (rate ratio = 1.73), as were those who reported sexual activity with someone they had just met (rate ratio = 1.39). Social media and dating application use were not significantly associated after adjustment.

**Conclusion:**

Risky sexual behaviors are highly prevalent among university students in Lebanon. These findings highlight the urgent need for culturally adapted educational interventions addressing substance use and promoting safer sexual behaviors among young adults in the Middle East.

## Introduction

Risky sexual behaviors (RSBs) among adolescents and young adults represent a major global public health concern. RSBs are commonly defined as behaviors that increase the likelihood of negative sexual health outcomes, including unprotected sex, multiple or concurrent sexual partners, sexual activity under the influence of alcohol or drugs, and engaging in transactional sex (1). These behaviors contribute substantially to the risk of sexually transmitted infections (STIs), HIV, unintended pregnancies, and abortions (1). Additionally, early sexual debut is often associated with other risk behaviors such as smoking and alcohol abuse, further escalating health risks (2–5).

The global burden of STIs highlights the magnitude of these concerns. According to the World Health Organization (WHO), more than 1 million STIs are acquired every day, with an estimated 374 million new cases of curable STIs annually among individuals aged 15–49 years (6). Nearly half of these infections occur among individuals aged 15 to 24, underscoring the vulnerability of this population (7). University students are particularly at risk due to factors such as increased sexual experimentation, exposure to peer influences, substance use, and limited access to confidential sexual health services (8–14).

Digital media has increasingly become intertwined with sexual behavior among youth. Online sexual interactions, including sexting, pornography consumption, and use of dating or hookup applications such as Tinder and Grindr, have become more frequent and normalized on university campuses (15). Research suggests that greater exposure to sexual content online is associated with earlier sexual initiation, unprotected sex, an increased number of partners, and higher rates of STIs (15–18).

In the Middle East, cultural conservatism and sexual taboos often limit access to accurate sexual health information and hinder open communication, including with healthcare professionals. Lebanon, a small developing country in the region, has a predominantly young population, with 44% under 24 years old (19). While social attitudes are gradually shifting toward more liberal views on sexuality (20), sexual activity, especially among females, remains stigmatized, risking underreporting and delayed care-seeking for STIs (21). Existing studies in Lebanon show low condom use among university students and highlight associations between male gender, liberal attitudes toward sexuality, and substance use with increased sexual risk-taking (22, 23).

However, most prior research in Lebanon has focused on sexual attitudes or contraceptive practices, without examining the combined influence of substance use and digital media on risky sexual behavior. Moreover, earlier studies have primarily reported the prevalence of RSBs but have not assessed the count of risky behaviors, which better reflects the intensity of risk-taking (22, 24).

Therefore, this study aims to investigate the prevalence and count of RSBs among students at a private university in Lebanon and to identify sociodemographic, behavioral, and multimedia-related factors associated with a higher number of risky behaviors in this unique cultural setting.

## Methodology

### Study design and setting

This study utilized a cross-sectional online survey to assess the prevalence and count of RSBs and associated factors among students at a private university in Lebanon. An anonymous self-administered online questionnaire was sent via email three times to students enrolled at the Lebanese American University (LAU) from all majors over a period of 7 months (from May 2022 to November 2022). The LAU is a large private university operating on two campuses, Beirut and Byblos, Lebanon, with over 8,000 registered students.

### Study population

Eligible participants were undergraduate and graduate students over 18 years old, currently enrolled in the university, and agreeing to the consent form.

### Sampling technique

The study is a cross-sectional survey-based study. A convenient sample of participants was recruited via a voluntary response sampling approach between May 2022 and November 2022. The Dean of Students sent an official email to all registered students at both campuses inviting them to participate. This was followed by a snowballing recruiting method, as the students were encouraged to participate in the study and to share the link widely among their peers via the university specific student WhatsApp groups and social media platforms (Facebook, LinkedIn, and Instagram).

### Sample size

Epi Info™ (Center for Disease Control, Atlanta, GA, USA. Available from: http://wwwn.cdc.gov/epiinfo) was used to determine the sample size required for investigating the prevalence and count of RSBs and associated factors among students enrolled in one university in Lebanon. This software was selected because it applies the single-population proportion formula within a validated, transparent interface that allows incorporation of finite population correction and design effect, ensuring reproducibility and accuracy of calculations. We considered a target population of 8,000 students currently enrolled at the time of the study in any of the Beirut or Byblos campuses of the LAU. The prevalence of RSBs in university students ranged considerably from 35 to 61% in previously published papers (25, 26). Given these findings, and based on existing literature and expert opinion, a conservative estimate of 50% prevalence of risky behavior was used to maximize the sample size, taking into account a 95% confidence interval (CI), a ±5% margin of error. The base SRS estimate was n0 = (Z2 p(1 − p)) /d2 = 384 students approximately needed as a minimum sample size. We applied a design effect of 1.5 to account for clustering and dependencies introduced by voluntary response and snowball recruitment, inflating the simple random sample size estimate from 383 to around 575.

### Data collection tool

A structured, self-administered questionnaire was developed specifically for this study based on existing literature and previously validated sexual questions from the Home, Education/Employment, Eating, Activities, Drugs, Sexuality, Suicidal Ideation and Safety (HEEADSSS) framework (27). The questionnaire was designed to collect information on sociodemographic characteristics (e.g., age, gender, year of study), sexual history, and engagement in specific. Sexual activity was defined as having engaged in consensual sexual activity with another person within the past 12 months. Participants who indicated that they were not sexually active were automatically skipped and did not complete the sexual behavior section of the questionnaire. The questionnaire also collected information on media use, defined as the self-reported frequency and type of engagement with digital platforms, including search engines, social media applications, dating applications, and online communication tools such as WhatsApp. Participants were asked to choose one answer out of multiple options listed (except for age) for every question. The instrument was piloted with a small group of students (*n* = 10) to ensure the clarity and relevance of the questions.

### Risky sexual behaviors

Risky sexual behaviors were defined as (1) having unprotected sex (anal, vaginal, or oral), (2) having two or more sexual partners at the same time, (3) having sex under the effect of drugs, (4) having sex under the effect of alcohol, (5) paying/getting paid for sex (1, 28–32). Each criterion was assigned a score of 1, and a composite score was computed as the sum of RSBs exhibited by each participant. The total score ranged from 0 (no RSBs) to 5 (engagement in all identified RSBs). The presence of any RSB (score ≥1) was used to estimate the prevalence of RSBs, while the count of RSBs (ranging from 0 to 5) was used as the dependent variable in the regression analysis to examine factors associated with a higher number of RSBs.

### Ethical considerations

The study was approved by the Institutional Review Board at the Lebanese American University under code LAU. SOM. RS1.28/Mar/2022 on 03/28/2022. Before filling out the online survey, participants were briefed about the study objectives and their right to withdraw at any time. An IRB-approved written informed consent form was obtained electronically prior to participation in the survey. To encourage honest reporting of sensitive behaviors, participants were assured of their anonymity, and no personally identifying information was collected. Participants were informed that their responses would be kept confidential and that they could withdraw at any time without penalty. Participants who indicated emotional distress due to the nature of the questions were provided with information about university counseling services in the consent form. Collected data were encrypted, stored in password-protected computers, and presented as de-identified electronic files in Microsoft Excel and SPSS. Only researchers in the team had access to the collected data.

### Data analysis

Descriptive statistics were conducted, with absolute frequencies and percentages presented for categorical variables and means and standard deviations (SD) for continuous quantitative measures. Bivariate analyses were performed to assess associations between RSBs and participant sociodemographic characteristics, substance use behaviors, sexual history variables, and multimedia use. The Pearson’s Chi-squared test was employed to evaluate associations between categorical variables, with the Fisher’s exact test used as an alternative when expected cell counts were below threshold values. For continuous variables, the independent Student’s t-test was used to compare means between two groups, while ANOVA was used to compare means across multiple levels of RSB scores. When significant differences were observed in ANOVA, *post-hoc* tests were conducted to identify specific group differences.

As previously mentioned, the prevalence of RSB was defined as the presence of any RSB (≥1), while the count of RSB ranged from 0 to 5. A Poisson regression analysis was conducted to evaluate factors independently associated with the count of RSBs. This model is widely recommended for count outcomes in behavioral and epidemiological studies, particularly when the goal is to assess frequency or intensity of engagement in a given behavior (33), rather than simply predicting the presence or absence of RSB. This methods was deemed the most appropriate analystic choice since the dependent variable was the number of RSBs, ranging from 0 to 5. Independent variables included gender, GPA, sexual orientation, religion, school, smoking, alcohol use, drug use, history of sexually transmitted diseases, use of contraceptives, use of dating apps, use of social media to find sexual partners, experiences of being harassed online, harassing others online, exchanging messages, exchanging pictures, and engaging in sex with someone just met.

In the Poisson regression analysis, incident rate ratios (IRRs) were reported along with their 95% confidence intervals (CIs) and *p*-values to determine the strength and significance of the associations. The reference group for each categorical variable was specified and used to interpret the relative increase or decrease in the rate of exhibiting RSBs. Post-estimation diagnostics were conducted to validate model assumptions. Overdispersion was assessed by the Deviance/df (0.353) and Pearson Chi-Square/df (0.309), both <1, indicating no significant overdispersion and supporting the appropriateness of Poisson regression. The model’s overall statistical significance was confirmed by the Omnibus Likelihood Ratio test (χ^2^ = 68.733, df = 23, *p* < 0.001). Model fit was satisfactory, and collinearity diagnostics were performed to ensure the robustness of the regression model.

All analyses were performed using IBM SPSS Statistics (version 29.0, IBM Corp., Armonk, NY, USA). A two-sided *p*-value ≤0.05 was considered statistically significant.

## Results

### Baseline characteristics and sexual activity

A total of 588 university students participated in this study, with 39.5% male and 60.5% female (Table 1). The average age of participants was 19.8 years, and the majority were enrolled in the School of Arts and Sciences (46.1%). Most participants lived with their families (70.2%), and the predominant nationality was Lebanese (90.5%). Religious beliefs were varied, with 14.3% identifying as non-religious.

**Table 1:** Baseline characteristics of the initial study population.

Characteristic	All	Male	Female	*p*-value
Number (%)	588 (100)	232 (39.5)	356 (60.5)	
Age (avg, yrs), SD	19.8 (2.3)	19.9 (2.6)	19.7 (2.1)	0.523
School				<0.001
Architecture and design	43 (7.3)	8 (3.4)	35 (9.8)	
Arts and Sciences	271 (46.1)	94 (40.5)	117 (49.7)	
Business	133 (22.6)	67 (28.9)	66 (18.5)	
Engineering	75 (12.8)	45 (19.4)	30 (8.4)	
Health Sciences	66 (11.2)	18 (7.8)	48 (13.5)	
Living arrangement				0.098
Alone	175 (29.8)	60 (25.9)	115 (32.3)	
Family	413 (70.2)	172 (74.1)	241 (67.7)	
Living area				0.440
Greater Beirut area	212 (36.1)	78 (33.6)	134 (37.6)	
Mount Lebanon	295 (50.2)	124 (53.4)	171 (48.0)	
Periphery	81 (13.8)	30 (12.9)	51 (14.3)	
Nationality				0.569
Lebanese	532 (90.5)	212 (91.4)	320 (89.9)	
Other	56 (9.5)	20 (8.6)	36 (10.1)	
Religious beliefs				0.022
Christian	228 (38.8)	99 (42.2)	130 (36.5)	
Muslim	219 (37.2)	80 (34.5)	139 (39.0)	
Other	57 (9.7)	14 (6.0)	43 (12.1)	
Non-religious	84 (14.3)	40 (17.2)	44 (12.4)	
Sexual orientation				0.012
Heterosexual	468 (79.6)	197 (84.9)	271 (76.1)	
Other	120 (20.4)	35 (15.1)	85 (23.9)	
Relationship status				0.458
Single	418 (71.1)	169 (72.8)	249 (69.9)	
In a couple	170 (28.0)	63 (27.2)	107 (30.1)	
Smoking				0.198
No	382 (65.0)	158 (68.1)	224 (62.9)	
Yes	206 (35.0)	74 (31.9)	132 (37.1)	
Alcohol				0.884
No	269 (45.7)	107 (46.1)	162 (45.5)	
Yes	319 (54.3)	125 (53.9)	194 (54.5)	
Drugs				0.041
No	479 (81.5)	187 (80.6)	292 (82.0)	
Yes	70 (11.9)	35 (15.1)	35 (9.8)	
Prefer not to say	39 (7.0)	10 (4.3)	29 (81.)	
GPA^a^				0.269
Less than 3.0	147 (25.0)	66 (28.4)	81 (22.8)	
3.0–3.5	184 (31.3)	67 (28.9)	117 (32.9)	
3.5–4.0	257 (43.7)	99 (42.7)	158 (44.4)	
Sexually active				<0.001
No	355 (60.4)	118 (50.9)	237 (66.6)	
Yes	233 (39.6)	114 (49.1)	119 (33.4)	

Frequency and percentages presented by column. Independent student t-test used for comparison between gender. ^a^GPA is reported on a 4.0 scale.

Of the participants, 233 (39.6%) reported being sexually active (Table 2). Among them, the average age at first sexual encounter was 17.2 years, with males reporting a significantly lower average age of encounter than females (16.5 vs. 17.9 years, respectively, *p* < 0.001). Participants reported an average of 3.25 lifetime sexual partners, with males having significantly more partners than females (*p* < 0.001) (Table 2). Condom use was reported inconsistently, with 13.7% of participants never using condoms during sexual activity. Moreover, 18.0% of participants reported having a STI at some point.

**Table 2:** Reported sexual activity and behavior among sexually active participants.

Characteristic	All	Male	Female	*P-*value
Number (%)	233 (100)	114 (48.9)	119 (51.1)	
Age at first sexual encounter (Avg, years), SD	17.2 ± 2.6	16.5 ± 2.6	17.9 ± 2.5	<0.001
Sexual partners in lifetime, SD	3.25 ± 2.0	3.8 ± 2.0	2.8 ± 1.9	<0.001
Use of a condom				0.601
Never	32 (13.7)	18 (15.8)	14 (11.8)	
Once/Sometimes	97 (41.6)	48 (42.1)	49 (41.2)	
Often/Always	104 (44.6)	48 (42.1)	56 (47.1)	
Use of OCP*				----
No	92 (77.3)	---	92 (77.3)	
Yes	27 (22.7)	---	27 (22.7)	
Use of contraception (condom or OC)				0.364
No	30 (12.9)	17 (14.9)	13 (10.9)	
Yes	203 (87.1)	97 (85.1)	106 (89.1)	
Ever had an STI				0.226
No	191 (82.0)	97 (85.1)	94 (79.0)	
Yes	42 (18.0)	17 (14.9)	25 (21.0)	
Sex with someone you just met				<0.001
Never	134 (57.5)	51 (44.7)	83 (69.7)	
Once/Sometimes	72 (30.9)	44 (38.6)	28 (23.5)	
Often/Always	27 (11.6)	19 (16.7)	8 (6.7)	

SD, standard deviation. Data are mean ± SD [min-max] for quantitative variables or number (percent) for categorical.

Frequency and percentages presented by column. Independent student *t*-test used for comparison between gender. *In females only.

### Risky sexual behaviors

**Figure 1:**
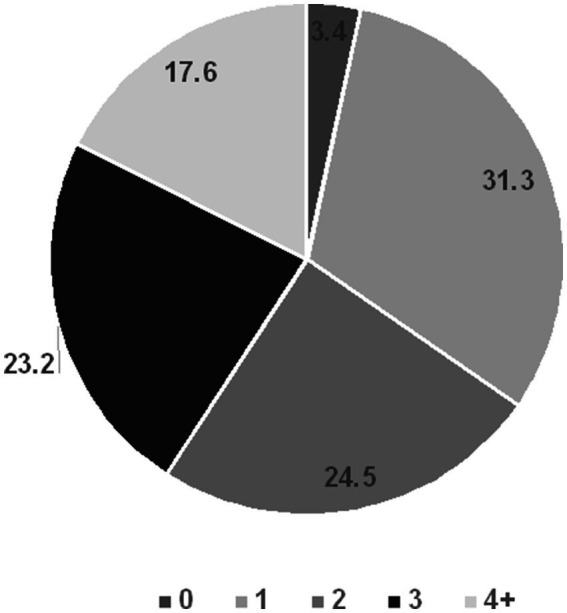
Average of risky sexual behaviors reported by sexually active university students in Lebanon, a Middle Eastern developing country.

Risky sexual behaviors were common, with almost all sexually active participants (225 of 233; 96.6%) reporting engagement in at least one RSB. The distribution of participants by the number of RSBs is shown in Figure 1.

**Figure 2:**
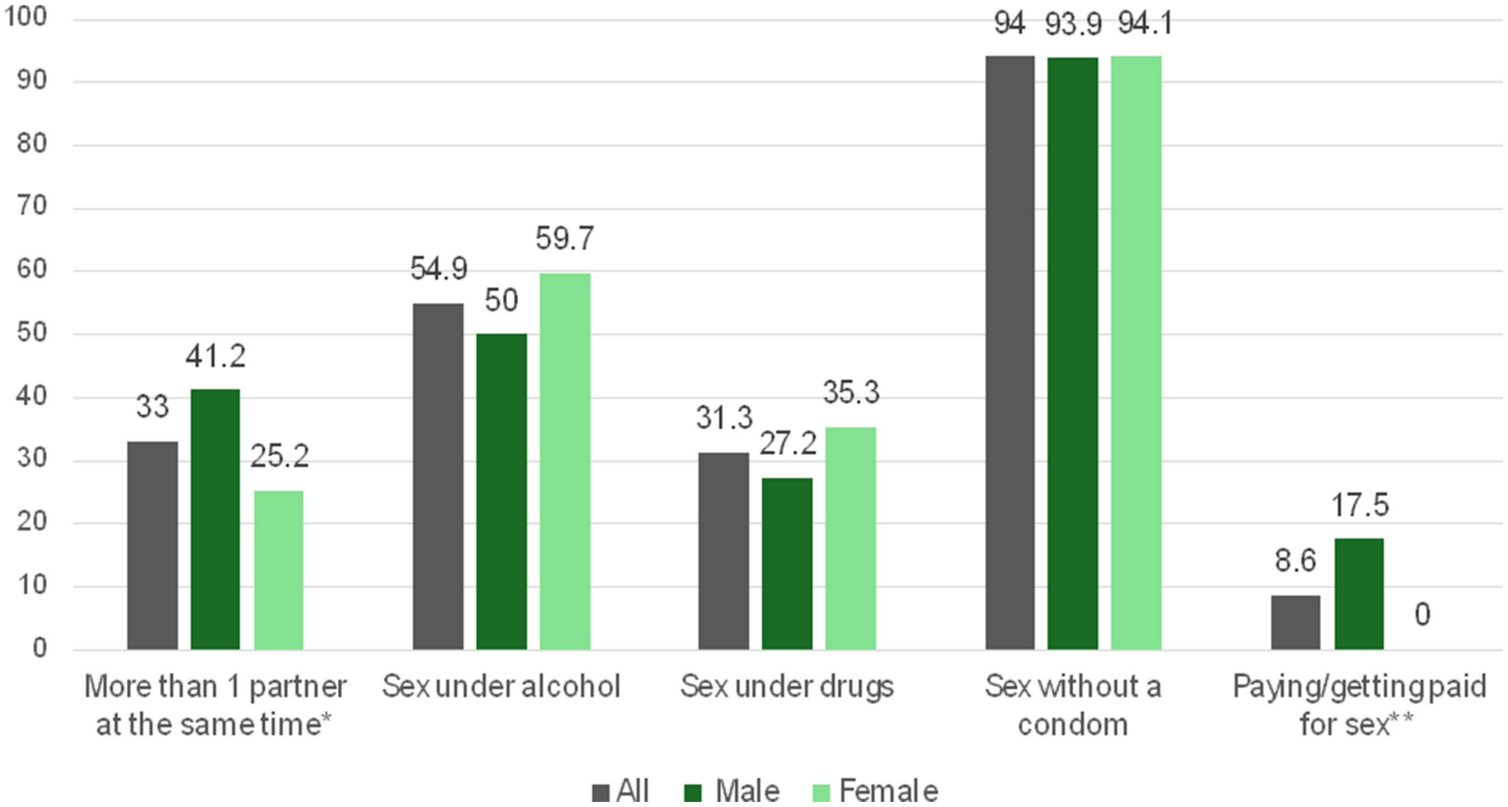
Reported frequency of risky sexual behaviors among sexually active participants, by gender. *p = 0.009; **p < 0.001.

In addition, the mean number of RSBs reported was 2.24 (SD = 1.25). Specifically, 33% reported having more than one partner at the same time, 54.9% reported having sex under the influence of alcohol, and 8.6% mentioned paying or getting paid in exchange for sex. Gender differences were observed, with males more likely to engage in multiple RSBs (Figure 2).

A breakdown of sociodemographic factors and behaviors associated with the number of RSBs among sexually active participants is presented in Table 3. Lower age at first sexual encounter, as well as behaviors such as smoking, drug use, and having sex with someone they just met, are all significantly associated with a greater number of RSBs (*p* < 0.001 for most). Interestingly, being in a relationship was associated with fewer RSBs, while being single correlated with a higher number of such behaviors (*p* < 0.001).

**Table 3:** Socio-demographic factors, substance use and other behaviors and their association with the average of risky sexual behaviors reported by sexually active participants.

Studied variables	Average of risky behaviors reported by sexually active students				
	1	2	3	4 or more	*P-*value
Number (%)	73 (32.4)	57 (25.3)	54 (24.0)	41 (18.2)	
Age at First sexual encounter	18.0 ± 2.7	17.4 ± 2.3	16.7 ± 2.6	15.9 ± 2.6	<0.001
Gender					0.792
Male	33 (30.0)	27 (24.5)	29 (26.4)	21 (19.1)	
Female	40 (34.8)	30 (26.1)	25 (21.7)	20 (17.4)	
School					0.090
Architecture and design	10 (47.6)	3 (14.3)	5 (23.8)	3 (14.3)	
Arts and Sciences	34 (36.6)	24 (25.8)	23 (24.7)	12 (12.9)	
Business	19 (29.2)	13 (20.0)	21 (32.3)	12 (18.5)	
Engineering	6 (25.0)	9 (37.5)	2 (8.3)	7 (29.2)	
Health Sciences	4 (18.2)	8 (36.4)	3 (13.6)	7 (31.8)	
Religion					0.019
Christian	29 (28.4)	36 (35.3)	21 (20.6)	16 (15.7)	
Muslim	24 (43.6)	8 (14.5)	15 (27.3)	8 (14.5)	
Other	4 (21.1)	4 (21.1)	3 (15.8)	8 (42.1)	
Non-religious	16 (32.7)	9 (18.4)	15 (30.6)	9 (18.4)	
GPA grouped^a^					0.189
Less than 3.0	16 (31.4)	10 (19.6)	16 (31.4)	9 (17.6)	
3.0–3.5	29 (40.8)	17 (23.9)	10 (14.1)	15 (21.1)	
3.5–4.0	28 (27.2)	30 (29.1)	28 (27.2)	17 (16.5)	
Sexual orientation					0.319
Heterosexual	53 (32.5)	38 (23.3)	44 (27.0)	28 (17.2)	
Other	20 (32.3)	19 (30.6)	10 (16.1)	13 (21.0)	
Relationship status					<0.001
Single	21 (19.6)	29 (27.1)	29 (27.1)	28 (26.2)	
In a Couple	52 (44.1)	28 (23.7)	25 (21.2)	13 (11.0)	
Smoking					<0.001
No	41 (44.6)	33 (35.9)	15 (16.3)	3 (3.3)	
Yes	32 (24.1)	24 (18.0)	39 (29.3)	38 (28.6)	
Alcohol					<0.001
No	28 (54.9)	11 (21.6)	8 (15.7)	4 (7.8)	
Yes	45 (25.9)	46 (26.4)	46 (26.4)	37 (21.3)	
Drugs					<0.001
No	66 (47.1)	47 (33.6)	22 (15.7)	5 (3.6)	
Yes	3 (5.1)	7 (11.9)	22 (37.3)	27 (45.8)	
Contraception use					0.495
No	10 (37.0)	6 (22.2)	4 (14.8)	7 (25.9)	
Yes	63 (31.8)	51 (25.8)	50 (25.3)	34 (17.2)	
Sex with someone they have just met					<0.001
No	59 (46.5)	37 (29.1)	25 (19.7)	6 (4.7)	
Yes	14 (14.3)	20 (20.4)	29 (29.6)	35 (35.7)	

Data are mean ± SD [min-max] for quantitative variables or number (percent) for categorical. Frequency and percentages presented by row. One-way Analysis of Variance (ANOVA) used for continuous variable (Age). Chi-square test used for categorical variables. ^a^GPA is reported on a 4.0 scale.

### Multimedia use for sexual purposes

Multimedia use for sexual purposes was common among participants, with 17.9% having used dating applications, 27.4% using social media to find sexual partners, and 57.7% watching pornography weekly. The use of multimedia for these purposes was significantly higher among males compared to females (Table 4). Furthermore, females were more likely to report being harassed or blackmailed online with sexual/explicit material (*p* < 0.001). Additional analysis revealed that among sexually active participants, 29.2% of males and 19.3% of females used dating applications, while 46.5% of males and 16.8% of females used social media to find sexual partners.

**Table 4:** Use of multimedia for sexual activity purposes among all students.

Characteristics	All	Male	Female	*P-*value
	*N* (%)	*N* (%)	*N* (%)	
	588 (100)	232 (39.5)	356 (60.5)	
Dating applications use				<0.001
Never	483 (82.1)	168 (72.4)	315 (88.5)	
Ever	105 (17.9)	64 (27.6)	41 (11.5)	
Social media use to find sexual partners				<0.001
Never	427 (72.6)	140 (60.3)	287 (80.6)	
Ever	161 (27.4)	92 (39.7)	69 (19.4)	
Duration of pornography exposure per week				<0.001
Never	249 (42.3)	51 (22.0)	198 (55.6)	
Less than 1 h	239 (40.6)	111 (47.8)	128 (36.0)	
Between 1–4 h	75 (12.8)	52 (22.4)	23 (6.5)	
5 h or more	25 (4.3)	18 (7.8)	7 (2.0)	
Ever been blackmailed/harassed online with sexual/explicit material				<0.001
No	476 (82.8)	208 (91.2)	268 (88.2)	
Yes	99 (17.2)	20 (8.8)	79 (22.8)	
Ever blackmailed/harassed someone online with sexual/explicit material				0.037*
No	579 (99.0)	225 (97.8)	354 (99.7)	
Yes	6 (1.0)	5 (2.2)	1 (0.3)	
Ever send /receive explicit messages of sexual content on social applications				0.569
No	220 (38.5)	89 (39.9)	131 (37.5)	
Yes	352 (61.5)	134 (60.1)	218 (62.5)	
Ever send/receive explicit pictures of sexual content on social applications				0.801
No	264 (46.2)	101 (45.5)	163 (46.6)	
Yes	308 (53.8)	121 (54.5)	187 (53.4)	

*Fisher exact test used, Chi-square test for the others. Frequency and percentage presented by column.

### Association between multimedia use and risky sexual behaviors

The relationship between the use of multimedia for sexual purposes and the number of RSBs is shown in Table 5. Participants who had ever used dating applications or social media to find sexual partners were significantly more likely to report a higher number of RSBs (*p* < 0.001 and *p* = 0.004, respectively). Those who had been harassed or had harassed someone online with sexual/explicit material were also more likely to engage in a higher number of RSBs although this association was not statistically significant in most cases.

**Table 5:** Percentages of students’ multimedia use for sexual activity purposes and their association with the average of risky sexual behaviors among sexually active participants.

Count of risky sexual behaviors	1	2	3	4 or more	*P-*value
Characteristics	*N* (%)	*N* (%)	*N* (%)	*N* (%)	
	73 (32.4)	57 (25.3)	54 (24.0)	41 (18.2)	
Dating applications use					<0.001
Never	59 (37.6)	45 (28.7)	33 (21.0)	20 (12.7)	
Ever	14 (20.6)	12 (17.6)	21 (30.9)	21 (30.9)	
Social media use to find sexual partners					0.004
Never	59 (38.1)	41 (26.5)	35 (22.6)	20 (12.9)	
Ever	14 (20.0)	16 (22.9)	19 (27.1)	21 (30)	
Ever been blackmailed/harassed online with sexual/explicit material					0.543
No	60 (34.1)	48 (27.3)	41 (23.3)	27 (15.3)	
Yes	13 (31.0)	8 (19.0)	12 (28.6)	9 (21.4)	
Ever blackmailed/harassed someone online with sexual/explicit material					0.019*
No	72 (32.9)	56 (25.6)	54 (24.7)	37 (16.9)	
Yes	0 (0)	1 (25)	0 (0)	3 (75)	
Ever Send/receive explicit messages on social applications					0.131
No	15 (41.7)	12 (33.3)	7 (19.4)	2 (5.6)	
Yes	58 (32.0)	43 (23.8)	45 (24.9)	35 (19.3)	
Ever Send/receive explicit pictures on social applications					0.144
No	23 (44.2)	14 (26.9)	10 (19.2)	5 (9.6)	
Yes	50 (29.9)	42 (25.1)	42 (25.1)	33 (19.8)	

### Multivariable analysis

The multivariable Poisson regression analysis (Table 6) revealed several factors associated with increased number of RSBs. Students who reported drug use had a 72.5% higher rate of RSBs compared to those who did not (IRR = 1.725, 95% CI: 1.338–2.223, *p* < 0.001). Additionally, engaging in sex with someone they had just met was associated with a 39.1% increase in the number of RSBs (IRR = 1.391, 95% CI: 1.084–1.783, *p* = 0.009). The use of alcohol approached significance, indicating a potential 30.6% increase in RSBs (IRR = 1.306, 95% CI: 0.973–1.754, *p* = 0.055). Although the use of dating applications and social media for sexual purposes showed an increase in the number of RSBs, these associations were not statistically significant after adjusting for other factors. Interestingly, no significant associations were observed between gender, GPA, sexual orientation, or religion and the number of RSBs.

**Table 6:** Multivariable analysis revealing variables associated with an increased number of risky sexual behaviors.

Studied variables	Adjuted incidence rate ratio (IRR)	95% confidence interval		*P-*value
		Lower bound	Upper bound	
Gender (female vs male*)	0.940	0.745	1.186	0.603
GPA (>3.5 vs ≤3.5*)^a^	0.984	0.797	1.216	0.884
Sexual orientation (Other vs heterosexual*)	0.916	0.717	1.172	0.487
Smoking (Yes vs no*)	1.087	0.836	1.414	0.534
Alcohol (Yes vs no*)	1.306	0.973	1.754	0.055
Drugs (Yes vs no*)	1.725	1.338	2.223	<0.001
Use of contraceptive (Yes vs no*)	1.193	0.866	1.642	0.280
Sexually Transmitted Disease (Ever vs never*)	0.897	0.689	1.168	0.420
Using dating applications (Ever vs never*)	1.065	0.817	1.389	0.640
Use of social media to find sexual partners (Ever vs never*)	0.977	0.754	1.267	0.863
Ever been harassed online (Yes vs no*)	0.933	0.704	1.239	0.633
Ever harassed someone online (Yes vs no*)	1.909	0.917	3.970	0.084
Ever Send /receive explicit messages on social applications (Yes vs no*)	0.951	0.635	1.423	0.806
Ever Send /receive explicit pictures on social applications (Yes vs no*)	1.241	0.852	1.808	0.260
Sex with someone just met (Yes vs no*)	1.391	1.084	1.783	0.009
Religion				
Non-religious	Ref			
Christian	1.045	0.786	1.389	0.762
Muslim	1.095	0.780	1.537	0.602
Other	1.103	0.734	1.658	0.635
School				
Health sciences	Ref			
Architecture and design	0.959	0.583	1.577	0.868
Arts and Sciences	0.954	0.680	1.337	0.785
Business	1.020	0.720	1.445	0.912
Engineering	1.128	0.737	1.727	0.579

Model: Poisson regression; dependent variable: number of risky sexual behaviors; independent variables: gender, GPA, sexual orientation, religion, school, smoking, alcohol, drugs, sexually transmitted disease, use of contraceptive, use of dating apps, use of social media to find sexual partners, harassed online, harass someone online, exchange messages, exchange pictures, sex with some just met. ^*^Reference group. ^a^GPA is reported on a 4.0 scale.

## Discussion

This study found a high prevalence of RSBs among university students in Lebanon, with substance use being the most frequently associated factor. While our findings are consistent with existing literature from other countries, they are particularly significant because new emergent variables related to media use and dating apps for sexual activities were studied in Lebanon, a Middle Eastern country often perceived as conservative. This provides new insights into sexual health behaviors in the region, challenging assumptions and underlining the importance of addressing these behaviors in a culturally diverse context.

Unprotected sex has been extensively studied, particularly in relation to its association with STIs and unintended pregnancies (34–38). Our findings revealed that “oral sex without a condom” is the most commonly reported RSB. This aligns with previous research highlighting low rates of condom use among Lebanese university students, where only 36% reported consistent condom use during sexual activity (22).

Our study is one of the first in the region to explore the relationship between multimedia usage and RSBs. Specifically, we found that individuals using dating applications for sexual purposes are significantly more likely to engage in RSBs. Notably, one-third of these users reported engaging in three or more risky behaviors during their lifetime. This pattern may be influenced by frequent exposure to sexual content across various media platforms, which can shape attitudes and normalize risky sexual practices. Such normalization may desensitize individuals, increasing their likelihood of engaging in RSBs (39, 40).

Social media has been discussed as being a source of sexual education among adolescents by shaping their understanding of sexuality and influencing their decisions regarding sexual activities, with its excessive use promoting risky behaviors (41). This is supported by a study that studied the impact of frequent exposure to online sexual content among youth, demonstrating that it can lead to increased RSBs and distorted perceptions of sexual relationships (42). The study highlights the idea that social media platforms can amplify peer pressure, normalizing RSBs through shared content and interactions, thereby influencing users’ attitudes and behaviors regarding sexuality.

A study conducted in the United States mid-Atlantic region underscores the growing prevalence of mobile dating apps and their association with RSBs among young adults. The study highlights how casual relationships facilitated by these apps often lead to impulsive behaviors, including unprotected sex and having multiple partners simultaneously (43). These behaviors are further exacerbated by the sense of anonymity and reduced accountability in online environments, which embolden individuals to engage in riskier behaviors compared to traditional social settings. This phenomenon can be attributed to easier access to potential partners as the necessity for face-to-face interactions is diminished. As a result, individuals may feel emboldened to engage in behaviors they might otherwise avoid in traditional social settings (44, 45). Although prior literature has demonstrated associations between media exposure and RSBs, this relationship was not significant in our adjusted model. A possible explanation is that the association between media use and RSBs in our sample may be indirectly expressed through behavioral factors such as drug use and sex with someone just met, which showed the strongest associations in the model (IRR = 1.725 and IRR = 1.391, respectively). When these behavioral variables were included, the effect of dating app use and social media use became statistically non-significant, suggesting that media use alone may not directly increase RSBs unless accompanied by actual behavioral risk-taking opportunities or situations. Furthermore, dichotomizing media use into “ever” vs. “never” may have limited our ability to detect frequency- or exposure-based effects, as occasional and frequent users were clustered together.

Alternatively, our findings reveal that 75% of individuals who reported engagement in four or more RSBs admitted to blackmailing or harassing others online using explicit material. Among those targeted by such harassment, females were disproportionately affected (22.8% compared to 8.8% for males). While this observed disparity should be interpreted with caution given the overrepresentation of females in our sample because of convenience sampling, yet relative to the university’s student population (55% of university wide students are female), it remains consistent with a multi-country Arab report by the United Nations on cyber violence, which indicates that women are more likely to experience online harassment than men (46, 47). This vulnerability may be reinforced by social norms in Lebanon, where women’s sexuality is more heavily scrutinized, and reputational harm carries greater consequences for females than males (48, 49). Such norms grant men greater social permission to engage in diverse sexual behaviors, while women face stigma and surveillance over their sexual choices.

Patriarchal structures in the Middle Eastern and North African (MENA) region has historical roots in tribal systems that prioritize male inheritance and leadership, where conservative interpretations of religion and cultural norms emphasize male dominance and control over women’s sexuality (48, 50, 51). Religious beliefs reinforce these structures by promoting strict gender roles and limiting women’s autonomy (49). Family dynamics and inheritance laws that favor male heirs exacerbate this imbalance, while societal emphasis on honor often results in heightened control over women’s behavior, particularly regarding sexuality (50). In our study, males were more likely than females to report having multiple simultaneous sexual partners, which is a well-recognized component of RSB globally and within the Middle East (52–54). Furthermore, our study found that only male participants reported paying for or receiving payment for sex, a behavior that significantly increases their risk of acquiring STIs (55). This discrepancy can be attributed to entrenched gender norms and double standards within Middle Eastern societies (56). Women may avoid disclosing such behaviors due to fear of being perceived as “easy” or “immoral” (56).

Religiosity also plays a significant role in shaping sexual behaviors. A 2023 meta-analysis revealed that formal religiosity is significantly associated with delayed age at sexual debut and fewer sexual partners (57). Similarly, our study found that identifying as Muslim or Christian correlated with lower levels of RSB among participants. This finding aligns with prior research conducted in Lebanon, which demonstrated that religious individuals exhibit reduced engagement in risky sexual practices (58).

Our study reveals significant associations between RSBs among university students in Lebanon and factors such as drug consumption and engaging in sexual activities with newly met individuals. Alcohol use was weakly associated with RSBs. These findings are consistent with broader research indicating that substance use can impair judgment, leading individuals to underestimate the risks associated with spontaneous and unplanned sexual activities (59–61). The impairment caused by alcohol and drugs can reduce inhibitions and increase the likelihood of engaging in risky behaviors, such as unprotected sex or having multiple sexual partners (59–63). A previous cross-sectional study conducted in Lebanon found that RSBs are nearly three times more likely to occur under the influence of alcohol or drugs (58). Similarly, research has shown that increased substance use is associated with a higher likelihood of engaging in sexual activity, an increase in the number of sexual partners, early sexual initiation, multiple sexual partners, unprotected sex, and unintended pregnancies (64–68). These findings underscore the need for targeted interventions focusing on substance use and sexual health education to mitigate the risks associated with these behaviors among university students.

In Lebanon, several socio-cultural factors contribute to substance use among young adults, which in turn increases their likelihood of engaging in RSBs. The economic crisis, weakened family supervision, and shifting social norms have contributed to increased alcohol and drug use as coping mechanisms for stress and uncertainty (23). Peer pressure also plays a significant role, particularly among young men who may feel socially encouraged to experiment with substances and pursue sexual opportunities as expressions of masculinity and social status (22). Moreover, the normalization of alcohol consumption and smoking in Lebanese society, especially among males, reduces perceived harm and facilitates situations where sexual disinhibition can occur (69). These patterns may partially explain our findings that substance use strongly predicts higher RSB counts, aligning with regional research demonstrating that substance-related sexual debut is associated with multiple partners and lower condom use (23).

Our finding that participants who engaged in sexual activity with someone they had just met were more likely to exhibit multiple RSBs is consistent with previous literature (67). This highlights the need for educational interventions that promote communication, consent, and safer decision-making in new sexual relationships.

While this study provides valuable insights into the prevalence, count, and factors associated with RSB among university students in Lebanon, there are several limitations that must be considered. First, the study employed a cross-sectional design, which limits our ability to establish causal relationships between RSBs and the associated factors. While we can identify correlations, the temporal direction of these associations remains unclear. Second, the use of self-reported data introduces the potential for social desirability bias, where participants may underreport or overreport certain behaviors, particularly those related to sensitive topics such as sexual activity and substance use. Despite efforts to ensure anonymity and confidentiality, participants may still have been reluctant to disclose behaviors perceived as socially undesirable. Additionally, the voluntary response sampling method may limit the generalizability of the findings. In this technique, participants self-select to respond to the survey, meaning only those interested in the topic or those motivated will complete it. This could have led to selection bias, as participants who chose to engage with the survey may differ from those who did not in ways that could influence the results. Furthermore, the study was conducted at a single private university in Lebanon, limiting its external validity to other universities or regions within the country or broader Middle Eastern context. The specific demographic characteristics of the study sample, including a predominance of Lebanese students and those from the School of Arts and Sciences, may not fully represent the entire student population at LAU or other institutions. As previously discussed, some variables, such as dating app use and social media use to find partners, were dichotomized into “ever” versus “never” due to highly skewed response distributions. While this approach preserved statistical power, it may have masked potential dose–response effects and limited the ability to detect frequency-related associations. Similarly, the study measured only religious affiliation and did not assess the degree of religiosity or engagement in religious practices, which may better reflect individuals’ values and behavioral norms. Future research should incorporate validated measures of religiosity to more accurately examine its influence on sexual behavior. Lastly, while the survey included a comprehensive set of variables related to RSBs and potential associated factors, other unmeasured factors, such as mental health status, peer influences, and detailed socio-economic background, could also contribute to RSBs. Future studies should aim to address these factors and employ longitudinal designs to better understand the long-term impacts and causality of these behaviors. Moreover, given the complex nature of sexual behavior, interdisciplinary approaches that incorporate perspectives from psychology, sociology, and public health are recommended to strengthen future research and interventions.

## Conclusion

This study demonstrates a high prevalence and count of RSBs among students at a private university in Lebanon, where shifting social norms may facilitate greater sexual risk-taking despite persistent cultural taboos. Substance use, particularly drug consumption, and engaging in sexual activity with newly met individuals were the strongest contributors to higher numbers of risky behaviors. These findings highlight the need for culturally tailored interventions aimed at Lebanese university students that focus on reducing substance-related sexual risk, promoting safer sexual decision-making, and addressing harm associated with online interactions. Future research should include a more diverse representation of students across different types of universities in Lebanon to enhance generalizability and better inform national sexual health strategies.

## Data Availability

The original contributions presented in the study are included in the article/Supplementary material, further inquiries can be directed to the corresponding author.
