# Supplementary material for: Prevalence of risky sexual behaviors and associated factors among students at a private university in Lebanon
Source: Front Public Health. 2025 Dec 19;13:1678926. doi: 10.3389/fpubh.2025.1678926 (PMC12757371; doi:10.3389/fpubh.2025.1678926)
Supplement: Supplementary file 1 [file Data_Sheet_1.pdf]

Consent to participate in a questionnaire. Factors associated with risky sexual behavior and STI treatment-seeking behaviors among young adults: A cross sectional study from Lebanon

I would like to invite you to participate in a research project by completing the following questionnaire/ survey. The purpose of this questionnaire / survey aims to evaluate risky sexual behaviors among university students and the factors associated with it.

There are no known risks, harms or discomforts associated with this study beyond those encountered in normal daily life. The information you provide will be used to enhance and improve our understanding of factors associated with risky sexual behaviors among university students . You will not directly benefit from participation in this study. The study will involve around 400 participants. Completing the survey will take 20 minutes of your time. Please feel free to share the link with your colleagues at the university.

By continuing with the questionnaire / survey, you agree with the following statements:

1. I have been given sufficient information about this research project.
2. I understand that my answers will not be released to anyone and my identity will remain anonymous. My name will not be written on the questionnaire nor be kept in any other records.
3. When the results of the study are reported, I will not be identified by name or any other information that could be used to infer my identity. Only researchers will have access to view any data collected during this research however data cannot be linked to me.
4. I understand that I may withdraw from this research any time I wish and that I have the right to skip any question I don't want to answer.
5. I understand that my refusal to participate will not result in any penalty or loss of benefits to which I otherwise am entitled to.
6. I have been informed that the research abides by all commonly acknowledged ethical codes and that the research project has been reviewed and approved by the Institutional Review Board at the Lebanese American University
7. I understand that if I have any additional questions, I can ask the research team listed below.
8. I have read and understood all statements on this form.
9. I voluntarily agree to take part in this research project by completing the following survey/Questionnaire.

If you have any questions, you may contact:
Name (PI) Phone number Email address

Rania Sakr, MD 03 461710 Rania.sakr01@lau.edu.lb
Youssef Rizk, MD 70 118476 Youssef.rizk@lau.edu.lb

If you have any questions about your rights as a participant in this study, or you want to talk to someone outside the research, please contact the:

Institutional Review Board Office,
Lebanese American University
3rd Floor, Dorm A, Byblos Campus
Tel: 00 961 1 786456 ext. (2546)
irb@lau.edu.lb

This study has been reviewed and approved by the LAU IRB:

* Indicates required question

1. Age

____

2. Gender *

Mark only one oval.

- Male
- Female
- Other

3. Major / Faculty *

Mark only one oval.

- Business
- Arts and Sciences
- Architecture and Design
- Medicine
- Pharmacy
- Nursing
- Engineering

4. Where are you living? *

Mark only one oval.

- With Family
- Alone in Dorms
- Alone in off-campus residence

5. In which area are you living? *

Mark only one oval.

- Greater Beirut Area
- Mount Lebanon (Baabda - Metn - Keserwan - Jbeil)
- Mount Lebanon (Aley - Chouf)
- North Lebanon
- South lebanon
- Bekaa

6. Nationality/ Hometown *

Mark only one oval.

- Lebanese
- Other Arab
- European
- North American
- South American
- Asian
- Austalia / Oceania

7. Religion *

Mark only one oval.

- Christian
- Muslim
- Druze
- Jewish
- Non-religious
- Other

8. Sexual Orientation *

Mark only one oval.

- Heterosexual
- Homosexual
- Bisexual
- Other

9. Relationship status *

Mark only one oval.

- Single
- Married
- In a relationship (including being engaged)

10. Do you smoke (Including cigarettes & hubble bubble) *

Mark only one oval.

- Yes
- No

11. Do you drink alcohol? *

Mark only one oval.

- Yes
- No

12. Do you use drugs? *

Mark only one oval.

- Yes
- No

13. What is your GPA? *

Mark only one oval.

- 3.5 – 4.0
- 3.0 - 3.5
- 2.5 – 3.0
- 2.0 – 2.5
- Less than 2

14. Are you sexually active? *

Mark only one oval.

- Yes *Skip to question 15*
- No *Skip to question 32*

Sexuality

15. Age at first sexual encounter? *

____

16. Number of Sexual partners in the last 12 months? *

Mark only one oval.

- 1
- 2
- 3
- 4
- 5 or more

17. If you are a female, do you use oral contraceptives or any other form of hormonal contraception? *

Mark only one oval.

- Yes
- No
- Not applicable (I am a male)

18. Do you use a condom? *

Mark only one oval.

- Yes
- No

19. Reason for not using a condom? *

Mark only one oval.

- Pressure/Being forced not to
- Financial reasons
- Lack of knowledge
- It is uncomfortable
- I trust my partner
- Not applicable (I always use condoms)

20. Frequency of screening for Sexually transmitted illnesses (STIs)/HIV? *

Mark only one oval.

- Never
- Once in a lifetime
- Once a year
- Once every 6 months or more

21. Frequency of having sex under the influence of Alcohol in the last 12 months? *

Mark only one oval.

- Never
- Once
- Sometimes
- Often
- Always

22. Frequency of having sex under the influence of drugs in the last 12 months? *

Mark only one oval.

- Never
- Once
- Sometimes
- Often
- Always

23. Frequency of performing oral sex without a condom in the last 12 months? *

Mark only one oval.

- Never
- Once
- Sometimes
- Often
- Always

24. Frequency of having vaginal sex without a condom in the last 12 months? *

Mark only one oval.

- Never
- Once
- Sometimes
- Often
- Always

25. Frequency of having anal sex without a condom in the last 12 months? *

Mark only one oval.

- Never
- Once
- Sometimes
- Often
- Always

26. Frequency of having sex with someone you just met in the last 12 months? *

Mark only one oval.

- Never
- Once
- Sometimes
- Often
- Always

27. Frequency of paying for sex or getting payed for sex in the last 12 months? *

Mark only one oval.

- Never
- Once
- Sometimes
- Often
- Always

28. Frequency of engaging in a sexual activity later regretted in the last 12 months? *

Mark only one oval.

- Never
- Once
- Sometimes
- Often
- Always

29. Frequency of using emergency pill ? (for females) *

Mark only one oval.

- Never
- Once
- Sometimes
- Often
- Always
- Not applicable (I am a male)

30. Did you ever get a Sexually transmitted illness (STI)? *

Mark only one oval.

- Yes
- No *Skip to question 32*

STD

31. 17. What Sexually transmitted illness (STI) did you get? *

Mark only one oval.

- Warts
- Pediculosis (Body itching)
- Chlamydia / Gonorrhea (discharge)
- Herpes
- Syphilis
- HIV /AIDS
- Other

Use of dating applications

32. Do you use dating applications? (Tinder, bumble, okcupid, grindr, badoo...etc..) *

Mark only one oval.

- Yes
- No

33. How often do you use dating applications (Tinder, bumble, okcupid, grindr, badoo...etc...) to find sexual partners? *

Mark only one oval.

- Never
- Once
- Sometimes
- Often
- Always

34. How often do you use social media applications (facebook, Instagram, snapchat, whatsapp...etc..) to find sexual partners? *

Mark only one oval.

- Never
- Once
- Sometimes
- Often
- Always

35. How many hours of pornography do you watch per week? *

Mark only one oval.

- Never
- Less than 1 hour
- Between 1 and 4 hours
- between 5 and 7 hours
- between 7 and 10 hours
- More than 10 hours

36. Were you ever Blackmailed/ harassed online with sexual/explicit material? *

Mark only one oval.

- Yes
- No

37. Did you ever Send / receive explicit messages on social applications? *

Mark only one oval.

- Yes
- No

38. Did you ever Send / receive explicit pictures on social applications? *

Mark only one oval.

- Yes
- No

Education / Knowledge about Sexually transmitted illnesses (STI)

39. If you ever got a sexually transmitted illness (STI), where did you seek treatment? *

Mark only one oval.

- Doctor
- Pharmacy
- Specialized sexual center
- Non profit Organization (NGO)
- Internet
- Parents
- Brothers/Sisters
- Friends
- Not applicable (I never had an STI)

This content is neither created nor endorsed by Google.

Google Forms
